# Perceived barriers and facilitators to infection prevention and control in Dutch residential care facilities for people with intellectual and developmental disabilities: a cross-sectional study

**DOI:** 10.1186/s12889-024-18159-9

**Published:** 2024-03-05

**Authors:** Famke Houben, Casper DJ den Heijer, Nicole HTM Dukers-Muijrers, Claudia Smeets-Peels, Christian JPA Hoebe

**Affiliations:** 1 Department of Social Medicine, Care and Public Health Research Institute (CAPHRI), Faculty of Health, Medicine and Life Sciences, Maastricht University, 6200 MD Maastricht, P.O. box 616, The Netherlands; 2 Department of Sexual Health, Infectious Diseases and Environmental Health, Living Lab Public Health Mosa, South Limburg Public Health Service, 6400 AA Heerlen, P.O. box 33, The Netherlands; 3 Department of Medical Microbiology, Infectious Diseases and Infection Prevention, Care and Public Health Research Institute (CAPHRI), Faculty of Health, Medicine and Life Sciences, Maastricht University Medical Centre (MUMC+), 6202 AZ Maastricht, P.O. box 5800, The Netherlands; 4 Department of Health Promotion, Care and Public Health Research Institute (CAPHRI), Faculty of Health, Medicine and Life Sciences, Maastricht University, 6200 MD Maastricht, P.O. box 616, The Netherlands; 5 Stichting Pergamijn, Mercator 2, 6135 KW Sittard, The Netherlands

**Keywords:** COVID-19, Infection control, Long-term care, Intellectual disability, Developmental disability, Cross-sectional studies

## Abstract

**Background:**

Adequate implementation of infection prevention and control (IPC) in residential care facilities (RCFs) for people with intellectual and developmental disabilities (IDDs) is crucial to safeguarding this vulnerable population. Studies in this field are scarce. This study aimed to identify perceived barriers to and facilitators of IPC among professionals working in these settings, along with recommendations to improve IPC, to inform the development of targeted interventions.

**Methods:**

We administered an online questionnaire to 319 professionals from 16 Dutch RCFs for people with IDDs (March 2021-March 2022). Perceived multilevel barriers and facilitators (guideline, client, interpersonal, organisational, care sector, and policy level) were measured on a 5-point Likert scale (totally disagree-totally agree). Recommendations were assessed using a 5-point Likert scale (not at all helpful-extremely helpful), supplemented by an open-ended question. Barriers, facilitators, and recommendations were analysed by descriptive statistics. Open answers to recommendations were analysed through thematic coding.

**Results:**

Barriers to IPC implementation included the client group (e.g., lack of hygiene awareness) (63%), competing values between IPC and the home-like environment (42%), high work pressure (39%), and the overwhelming quantity of IPC guidelines/protocols (33%). Facilitators included perceived social support on IPC between professionals and from supervisors (90% and 80%, respectively), procedural clarity of IPC guidelines/protocols (83%), and the sense of urgency for IPC in the organisation (74%). Main recommendations included the implementation of clear IPC policies and regulations (86%), the development of a practical IPC guideline (84%), and the introduction of structural IPC education and training programmes (for new staff members) (85%). Professionals also emphasised the need for IPC improvement efforts to be tailored to the local care context, and to involve clients and their relatives.

**Conclusions:**

To improve IPC in disability care settings, multifaceted strategies should be adopted. Initial efforts should involve clients (and relatives), develop a practical and context-specific IPC guideline, encourage social support among colleagues through interprofessional coaching, reduce workload, and foster an IPC culture including shared responsibility within the organisation.

**Supplementary Information:**

The online version contains supplementary material available at 10.1186/s12889-024-18159-9.

## Background

Institutional care settings such as residential care facilities (RCFs) for people with intellectual and developmental disabilities (IDDs) place residents at an increased risk of infection [1–5]. Individuals residing in these institutions often have underlying health conditions and compromised immune systems, making them more susceptible to infections [2–5]. In addition to host factors, facility factors such as grouped-living conditions and frequent close contact with healthcare workers (HCWs) may lead to a higher risk of onset and transmission of infectious diseases [1]. Notably, previous Dutch studies have underscored the significant impact of infectious diseases in disability care settings, for both residents and HCWs. During the COVID-19 pandemic, the attack rate within disability care was 92%, far higher than in other outbreaks [4]. Furthermore, another Dutch study indicated higher rates of COVID-19 seroprevalence among HCWs in disability care compared to other care sectors and the general population [6]. Therefore, infection prevention and control (IPC) is critical in such settings to minimise the spread of infectious diseases and promote the health and safety of residents and HCWs.

HCWs play a critical role in preventing the spread of infectious diseases by following appropriate IPC protocols, such as hand hygiene and the use of personal protective equipment. However, previous studies have demonstrated suboptimal compliance of HCWs with IPC practices in disability care settings [7, 8]. The urgency to improve IPC in the disability care sector has been underscored by the findings of a recent report by the Dutch Health Inspectorate [8], which has concluded that IPC implementation requires further improvements and called for urgent action to enhance IPC in this care setting.

While HCWs play a crucial role in preventing the spread of infectious diseases through individual behavioural changes [7, 9], successful IPC requires considering broader multilevel factors [10–12]. These factors — operating at micro, meso, and macro levels — encompass barriers and facilitators that play an important role in shaping the success of IPC efforts [11, 13–16]. At the micro level, client-related factors, such as a lack of patient education or non-compliance regarding IPC may come into play [16]. In addition, interpersonal factors, such as exemplary behaviour among colleagues and HCWs stimulating clients to implement adequate IPC practices can facilitate IPC [11]. At the meso level, facilities should have clear policies in place to guide IPC practices, such as continuous IPC training programmes [11, 12]. Furthermore, managerial support and organisational priority, as well as IPC material and resource availability, are vital in ensuring the sustainability of IPC programmes. At the macro level, care sector and policy factors may also influence IPC [11]. For instance, the prevailing values and professional norms within the disability care sector can shape the attitudes and behaviours of HCWs regarding IPC. Additionally, access to adequate funding and resources, legislation and regulatory frameworks, and public policies may impact IPC efforts [11]. Moreover, the work frame of IPC is often articulated in guidelines. Well-defined national guidelines and their implementation in facilities can provide the necessary structure and direction for successful IPC [11].

The effectiveness of IPC can be influenced by various factors on multiple levels that may hinder or enable IPC [11, 13, 15]. Identifying these potential barriers and facilitators as perceived by professionals, and incorporating recommendations from professionals can improve the effectiveness of IPC programmes, and increase engagement and commitment to IPC [17]. While there are substantial studies on IPC in hospital and nursing home settings, studies examining IPC in disability care settings are limited [7, 11]. Therefore, this study aimed to examine the perceived barriers to and facilitators of IPC among professionals in RCFs for people with IDDs on various levels, including the guideline, client, interpersonal, organisational, care sector, and policy level. This study also aimed to identify recommendations reported by professionals to improve IPC in these settings. The objective was to validate previous qualitative findings among a broader professional group and rank the perceived barriers and facilitators. The findings of this study can help develop more effective IPC strategies that address the specific challenges faced in RCFs for people with IDDs.

## Methods

### Study design and setting

This cross-sectional study involved administering questionnaires to HCWs in RCFs for individuals with IDDs. It formed a part of a larger mixed methods study (NIEZT, Needs assessment for infection prevention among healthcare professionals outside the hospital) (grant number: 331,618), which objective was to examine IPC compliance and its determinants among professionals from RCFs for people with IDDs in the Netherlands.

Although efforts have been made to improve IPC in the disability care sector, effective IPC implementation remains challenging for many facilities [7, 8]. Nevertheless, an increasing number of disability care facilities are motivated and willing to address this issue and optimise IPC implementation.

The diversity in the care sector regarding different professionals (non-medical vs. non-medical) and clients (ranging from clients with mild disabilities to clients with severe or profound disabilities) has been recognised to be a challenge for IPC [11, 18]. In addition, the care sector is characterised by diverse types of care provision, including residential care, ambulatory care, and assisted living care, which may also pose challenges regarding IPC [18].

### Participants

Participants consisted of professionals from 16 RCFs for people with IDDs located in the southern and western parts of The Netherlands. Our objective was to comprehensively examine multilevel barriers and facilitators to IPC implementation. Therefore, we sought the participation of professionals with various educational backgrounds and occupations, working at multiple layers within the organisation. We included HCWs and managerial and policy-related professionals. Managerial and policy professionals were included for their perspectives on policies, workflows, and funding and resource allocation, providing valuable insights into potential barriers and facilitators at the organisational or policy level. Administrative personnel, primarily engaged in administrative tasks, were excluded due to their limited exposure to clients and IPC dynamics.

### Recruitment and data collection

For this study, a convenience sampling approach was used to select participants. First, we contacted a contact person at each umbrella organisation in the disability care sector via email or telephone, explaining the study’s purpose and inviting voluntary participation. If the contact person agreed to participate, they were asked to distribute the online questionnaire among their organisation’s staff members via an online platform. As an incentive for participation in the study, facilities that obtained a minimum of 30 responses from their staff members were provided with a facility-specific report that ranked the perceived barriers, facilitators, and recommendations reported by professionals.

To ensure the questionnaire’s suitability, we consulted with experts in the disability care sector to obtain their input before distributing it to our participants. The questionnaire was piloted among disability care physicians and reviewed by infection control professionals. These experts confirmed the questionnaire’s applicability, and only minor modifications were made. Before starting the questionnaire, participants provided their consent online after being provided with information on the objectives and content of the study.

If no responses were received after two weeks of the initial request, we sent a reminder to the contact person of the respective organisation. The data collection period lasted from March 2021 to March 2022, during the COVID-19 pandemic (mainly Alpha and Delta variant periods).

### Measurement instrument and study variables

The questionnaire included 45-items regarding barriers to and facilitators of IPC on the guideline, client, interpersonal, organisational, care sector, and policy level. Although guidelines are typically designed at the national or policy level and implemented at organisational level, considering their specific characteristics is important to understand their impact on IPC practices. The included items were based on qualitative findings of our previous study in the disability care setting [11], and established theoretical frameworks including the MIDI checklist of Fleuren et al. [14], the TICD checklist of Flottorp et al. [15], and theories of Grol and Wensing on incentives and barriers to healthcare changes [13].

Participants were asked to indicate to what extent they perceived an item as a facilitator or barrier by responding on a 5-point Likert scale from 1 (“totally disagree”) to 5 (“totally agree”). In addition, an “I do not know” answer option was added, as we included a broad set of different professionals (HCWs, managerial, and policy-related professionals). Example questions included: “The guidelines/protocols clearly describe the IPC activities I should perform and in which order.” (procedural clarity, guideline level), “Clients will generally not cooperate if IPC should be applied.” (lack of client cooperation, client level), “I can count on adequate assistance from my colleagues if I apply IPC.” (perceived social support, interpersonal level), “High work pressure affects structural IPC application.” (high work pressure, organisational level), “The home-like environment poses dilemmas when applying IPC.” (competing values between IPC and the home-like environment, care sector level), “Government agencies, such as the National Institute for Public Health and the Environment (RIVM), have sufficient initiatives and policies in place that focus on IPC.” (policy efforts of governmental agencies, policy level). The direction of the formulation of items (i.e., whether a factor was a facilitator or barrier) was based on the qualitative findings of our previous study [11], and based on theories of Fleuren et al. [14], Flottorp et al. [15], and Grol and Wensing [13]. Furthermore, in order to mitigate response bias and maintain respondent engagement, the direction of certain questionnaire items was intentionally alternated. Moreover, some included items are specific methods to improve IPC and were therefore positively formulated to examine the extent to which these factors already existed. To identify additional perceived barriers and facilitators to IPC, an open-ended question was included. In addition to perceived barriers and facilitators, items on recommendations were included. These items on recommendations were based on existing behaviour change methods [19, 20], and previous qualitative findings [11]. Respondents were asked to rate the helpfulness of each recommendation on a 5-point Likert scale from 1 (“not at all helpful”) to 5 (“extremely helpful”). We also included an open-ended question to capture additional recommendations from professionals on how to improve IPC.

### Data analysis

We only used complete questionnaires for analysis. The data was analysed using descriptive statistics to determine the proportion of professionals who perceived each factor as a facilitator or barrier to IPC implementation. Response options “(dis)agree” and “totally (dis)agree” (score of 4 or higher on the 5-point Likert scale) were categorised as facilitator or barrier. To determine the proportion of professionals who perceived each factor as facilitator or barrier, we divided the number of professionals who scored 4 or 5 (for facilitators or barriers) by the total number of participants that answered the question. The results were reported as percentages and frequencies. The answer option “I do not know” was considered as missing data and not included in the frequency calculation. For the recommendations, the close-ended questions were also analysed by descriptive statistics. We calculated the percentages of participants who scored 4 or 5 (i.e., “helpful” or “extremely helpful”) on each item. The open answers to recommendations were analysed through thematic coding. Moreover, sensitivity analyses were conducted to assess potential differences in perceived barriers to and facilitators of IPC among different professional groups. All analyses were performed using IBM SPSS Statistics version 27, and *p* < 0.05 was considered statistically significant.

## Results

### Study population

Of the initial 20 approached facilities, responses were received from participants from 16 facilities (80%). Reasons for facilities not to participate included time constraints and staff shortages resulting from the substantial burden of COVID-19 cases and outbreaks in the facilities. In total, 323 participants completed the online questionnaire, of whom a total of 319 responses (98.8%) met the inclusion criteria. The characteristics of the participants are shown in Table 1. The majority of participants were women (86.5%), with a mean age of 44 (± 12.2 years). Approximately half of the participants were non-medical professionals (58.9%).

**Table 1:** Characteristics of participants (*n* = 319)

	% (n)/M (SD)
*Sex*	
Man	13.5 (43)
Woman	86.5 (276)
*Age*	44 (12.2)
*Professional group*	
Medical professionals ^a^	30.4 (97)
Non-medical professionals ^b^	58.9 (188)
Managerial and policy professionals ^c^	10.7 (34)

*Abbreviations.* M = mean, SD = standard deviation

^a^Medical professionals included physicians, nurses, medical assistants, nursing assistants, and paramedical professionals with physical contact (physiotherapist, dental hygienist, chiropodist)

^b^Non-medical professionals included social workers (e.g., personal care attendants) and behavioural specialists (i.e., psychologists, behavioural scientists, remedial educationalists, and coaches/therapists)

^c^Managerial and policy professionals included managers, supervisors, policy or quality assurance officers, and infection control professionals

### Perceived barriers and facilitators to IPC implementation

**Fig. 1:**
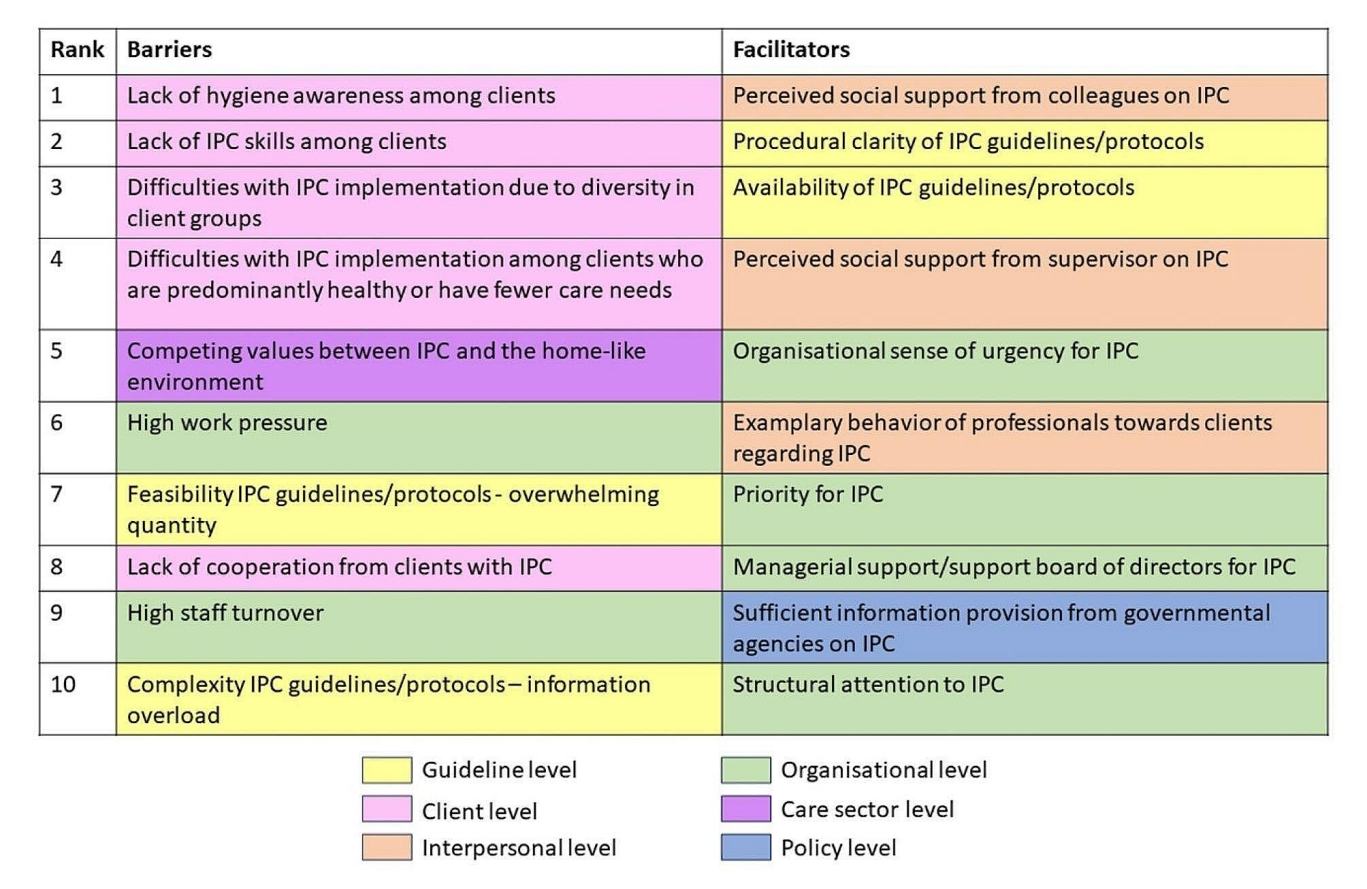
Perceived barriers to and facilitators of IPC according to professionals (*n* = 319) from residential care facilities (RCFs) for people with intellectual and developmental disabilities (IDDs), listed in rank order, along with their corresponding level: guideline (yellow), client (pink), interpersonal (orange), organisational (green), care sector (purple), and policy level (blue)

The perceived barriers and facilitators were categorised into the guideline, client, interpersonal, organisational, care sector, and policy level. Figure 1 shows a visual presentation of the rank order of the perceived barriers to and facilitators of IPC, along with their corresponding level. Additional file 1 presents an overview of the perceived barriers to and facilitators of IPC implementation, reported by professionals, and categorised by corresponding level. The majority of barriers to IPC implementation were reported at the client level, with the lack of hygiene awareness among clients (63.5%) and their lack of IPC skills (62.8%) being the most frequently perceived barriers. In addition, the diverse nature of client groups was perceived as a challenge to IPC implementation (61.9%). Furthermore, participants identified difficulties in implementing IPC practices among clients who are predominantly healthy or have fewer care needs (45.2%). Besides the client group, the second most perceived barrier was competing values between IPC and the home-like environment (42.1%), followed by high work pressure (38.9%), and an overwhelming quantity of IPC guidelines/protocols (32.9%). Facilitators of IPC implementation were also established. The most frequently reported facilitator was perceived social support from colleagues on IPC (89.7%). The second most commonly perceived facilitator was procedural clarity of IPC guidelines/protocols (83.1%), followed by availability of IPC guidelines/protocols (81.8%), perceived social support from supervisor on IPC (80.3%), and organisational sense of urgency for IPC (73.7%).

Apart from the rank order, additional information regarding potential facilitators can be identified. For this it is important to consider the nuanced aspects surrounding potential facilitators, recognising that their absence might pose hinderances. On the guideline level, only half of the professionals reported that the IPC guidelines/protocols are compatible with the workplace. In addition, 37.8% of participants reported that the IPC guidelines/protocols were adaptable (i.e., flexible to adjust or customise according to professional considerations). Regarding professional interaction, only half of the participants perceived sufficient interprofessional collaboration regarding IPC. Concerning the interaction between professionals and clients, 43.1% perceived sufficient stimulation and motivation of professionals towards clients. Furthermore, half of the participants reported sufficient feedback and accountability of professionals towards clients regarding IPC. On the organisational level, 27.6% of professionals perceived sufficient IPC education and training for clients, 42.5% for non-medical professionals, and 44.4% for new employees. Of the professionals, only 55.4% reported having a responsible professional for IPC (e.g., infection control professional) within their organisation. At the care sector level, 48.4% of participants perceived that IPC is a collective concern in the disability care sector. Regarding interorganisational collaboration, only 43.4% of professionals perceived sufficient collaboration between disability care facilities regarding IPC, and only half of the professionals perceived sufficient collaboration between disability care facilities and other health organisations such as the Public Health Service or hospitals. On the policy level, less than half of the professionals (47.1%) perceived sufficient governmental initiatives and policies focussing on IPC, and only half (51.2%) reported sufficient information provision from the professional association (NHG) on IPC.

### Sensitivity analyses for potential differences in perceived barriers and facilitators among different professional groups

Sensitivity analyses assessing potential differences in perceived barriers to and facilitators of IPC among different professional groups revealed similar results (data not shown).

### Recommendations reported by professionals to improve IPC

To explore recommendations reported by professionals, we provided possible strategies to improve IPC. Table 2 presents an overview of the recommendations to improve IPC, according to professionals, listed in rank order. The top five recommendations for improving IPC implementation reported by professionals were: (1) the need for clear policies and guidelines regarding IPC (85.7%); (2) the introduction of structural IPC training programmes for new employees (84.6%); (3) the development of a practical IPC guideline (84.3%); (4) organisation-wide IPC education and risk communication to raise awareness among both professionals and clients about the importance of IPC (79.9%); and (5) the inclusion of IPC as a structural part in the curriculum of both medical and social work educational programmes (78.5%).

**Table 2:** Recommendations to improve IPC (5-point Likert scale statements) according to professionals (*n* = 319) from residential care facilities (RCFs) for people with intellectual and developmental disabilities (IDDs), listed in rank order

Recommendations to improve IPC	proportion (n) of participants who indicated the recommendations as helpful (helpful-extremely helpful)
1. *Clear agreements/policies on organisational level* regarding IPC measures and guidelines.	85.7% (251)
2. Introduction of *structural IPC training for new employees.*	84.6% (248)
3. Development of a *practical IPC guideline*, tailored to disability care, including concrete task descriptions and short texts with images.	84.3% (247)
4. *Organisation-wide IPC education and risk communication* about the causes and consequences of infections, ensuring that all professionals and clients are made aware about the importance of IPC.	79.9% (234)
5. More *attention to IPC in basic educational programmes* (both in medical and social-work study programmes): IPC as a structural component of the curriculum.	78.5% (230)
6. Promotion of *organisation-wide information exchange and dissemination of educational materials on IPC*, ensuring that IPC information reaches all employees to enhance awareness and knowledge.	73.0% (214)
*7. Sharing information between disability care facilities* (e.g., during a sectoral meeting) to learn about what other organisations are doing regarding IPC and to identify challenges they face and how they address them.	72.0% (211)
8. Introduction of *structural IPC training for non-medical professionals.*	71.3% (209)
9. *Client participation*: increased involvement of clients in IPC, by for example providing more information and, if possible, offering IPC education to clients.	70.6% (207)
10. Introduction of *structural IPC training for all employees.*	70.3% (206)
11. *Appointment of a professional responsible for IPC* and coordinating its implementation, such as an infection control professional.	64.8% (190)
12. *Assessment and feedback*: assessing IPC behaviour of healthcare professionals and providing personalised recommendations to increase awareness of one’s own IPC practices.	60.8% (178)
13. *Widespread use of IPC reminders*, such as posters or reminder messages on online platform or via email.	59.7% (175)
14. *Increased coordination between departments and teams* through collaborative agreements, creating a clear separation and distribution of tasks and responsibilities regarding IPC.	58.7% (172)
15. Increased action from management; clear *IPC leadership*.	57.3% (168)
16. *Regular interprofessional knowledge-sharing and feedback* on IPC practices.	53.2% (156)
17. Allocate a *larger budget to IPC* within the organisation.	40.6% (119)
18. *Enhanced enforcement*: implementation of sanctions for IPC non-compliance by healthcare workers.	30.4% (89)

*Abbreviations.* IPC = infection prevention and control

In open-ended responses, as presented in Table 3, a major theme that emerged was the need for IPC improvement efforts to be tailored to the local care context. Professionals indicated the need to tailor IPC strategies to the diversity in the care sector and differentiate between different client groups (ranging from mild to severe and profound disabilities) and different types of care provision (residential vs. ambulatory care settings). A second major theme was the call for involving clients and their relatives in IPC. For this, professionals reported the specific recommendation to implement humorous educational and awareness campaigns and programmes for clients. Another theme that emerged was the need for local coaching and guidance regarding IPC in the team, for which professionals recommended appointing IPC contact persons and promoting open discussions regarding IPC challenges — and potential solutions — in team meetings. An additional theme was the need for sustained attention to IPC, for which organisations should also prioritise and pay attention to IPC in non-COVID-19 pandemic periods. Regarding specific IPC education and training strategies, professionals emphasised the importance of providing information on basic principles of infection transmission and control. They also highlighted the need to share tips and tricks for handling non-cooperation of clients with IPC, while emphasising the significance of ongoing and structural IPC education and training strategies.

**Table 3:** Recommendations to improve IPC (open-ended question), according to professionals (*n* = 319) from residential care facilities (RCFs) for people with intellectual and developmental disabilities (IDDs)

Themes	Key illustrative comments that reflect the answers on the theme
Tailor IPC strategies to diversity in the care sector and differentiate between different client groups and different types of care provision	“We are highly dependent on the behaviour of our clients in this regard. Often, they do not understand an IPC measure, and it conflicts with their rituals and support needs. Therefore, context-sensitive and tailored actions are needed.” “Within ambulatory care, we are guests in clients’ home environments. This can be problematic when the client follows a specific lifestyle and there is no room for IPC. This requires careful action.” “Customisation for different departments and groups is needed. Ambulatory staff require different rules than those working in residential care facilities.” “There is a distinction between residential and ambulatory care settings where the client’s network should be taken into account with regard to IPC.” “Segmenting client groups is necessary. Clients cannot be compared; client groups with mild intellectual disabilities are very different from groups with severe and profound disabilities. This demands differentiation and customisation, as well as awareness from the government.”
Involve clients and their relatives in IPC	“Providing client-centered information on the client’s level.” “Parents of clients should also be involved in this. Discuss with the team and parents what is desirable and necessary. We highly value a personalised approach.”
Local/team coaching and guidance on IPC	“Designating a person in the organisation who, for example, visits teams every three months to refresh their knowledge of IPC.” “Making it [IPC] a topic of discussion during team meetings, addressing challenges and exploring potential solutions.” “Emphasising coaching in the workplace, integrating IPC into daily practices for both clients and staff.” “Sensitisation within the group is important, maintaining a local approach.” “Appointing IPC contact persons within each facility or department.” “Having someone visit the residences to provide information and work together with the non-medical staff to identify and address their specific challenges in practice.”
Ongoing and structural attention to IPC (not only during the COVID-19 pandemic)	“Structural and ongoing attention is needed to IPC, not only when there is something like an epidemic or the current pandemic.”
IPC education/training	“Creating awareness by providing information on how infections are transmitted, as well as highlighting the dirtiness of our own hands using a lamp and identifying frequently touched areas in a department.” “More attention to and tips for clients who strongly resist IPC measures based on their level of understanding.” “Learning from and with each other. Professionals need to be aware that their behaviour and failure to use the appropriate measures can harm the client.” “Continuing education, possibly as part of earning accreditation points.”
Increase access to IPC materials and facilities	“Hang alcohol dispensers in more locations within our living group. This will encourage greater use of them.” “Ensure that there are always sufficient supplies available by proactively planning ahead and not falling behind on ordering.”
Increase staff resources	“More hands at the bedside. Too many vacancies; too few applicants.”
Modelling, setting a good example	“A good example sets a good precedent.”
Involve temporary workers	“Involve temporary workers strongly in IPC. I often see non-compliance among this group. They are resistant towards it [IPC].”
Outsource cleaning externally (no cleaning by clients)	“Better cleaning by domestic helpers and no clients being the cleaning lady! Delegate more household tasks to professional cleaning organisations, such as laundry and cleaning.”
Increase monitoring and feedback	“More monitoring, for example, during medication checks to examine the presence of artificial nails, which are very common among night shift caregivers.”

*Abbreviations.* IPC = infection prevention and control

## Discussion

This descriptive cross-sectional questionnaire study assessed perceived barriers to and facilitators of infection prevention and control (IPC) implementation in residential care facilities (RCFs) for people with intellectual and developmental disabilities (IDDs), along with recommendations reported by professionals to improve IPC, to inform targeted intervention development. The findings of our study indicated that barriers to IPC implementation included the client group (e.g., lack of hygiene awareness), competing values between IPC and the home-like environment, high work pressure, and an overwhelming quantity of IPC guidelines/protocols. Facilitators were perceived social support among colleagues on IPC, procedural clarity of IPC guidelines/protocols, and organisational sense of urgency for IPC. The main recommendations reported by professionals included clear IPC policies and regulations, a practical IPC guideline, and structural IPC education and training programmes (especially aimed at new staff members), while professionals also emphasised the need for IPC improvement efforts to be tailored to the local care context, and involve clients and their relatives. Our findings also indicated potential room for improvement regarding the enhancement of certain facilitators. IPC guidelines or protocols were not always compatible with the workplace, and professionals reported gaps in IPC education and training for clients, non-medical professionals, and new employees. In addition, interprofessional collaboration regarding IPC, and stimulation and motivation of clients by professionals were perceived as insufficient. Limited collaboration between disability care facilities and other health organisations, as well as insufficient governmental policy efforts on IPC, were also identified.

The findings of our study regarding barriers to and facilitators of IPC are in accordance with previous research conducted across different healthcare settings. Previous studies in other long-term care settings have acknowledged that the patient group can pose a challenge for IPC implementation [11, 21], for which a lack of awareness or knowledge regarding IPC were common patient-related barriers to IPC implementation. In addition, another study has indicated that patients with limited mobility or cognitive impairment were less likely to comply with IPC [22]. In line with the findings of the present study, previous studies have pointed to the influence of workplace culture on IPC implementation [12, 23]. Our previously conducted qualitative study in the disability care setting has indicated the challenge professionals face regarding perceived dilemmas between IPC and preserving a home-like environment [11]. Furthermore, studies conducted across long-term care settings have highlighted the problem of high workload and high work pressure in the workplace, which is recognised as a hindrance to adequate IPC implementation [11, 16, 24]. Moreover, previous studies have suggested that an overload of different IPC guidelines or protocols and the complexity of guidelines or protocols can be significant barriers to IPC implementation [11, 12], which aligns with the findings of our study. A previous Cochrane review conducted in other healthcare settings concluded that HCWs often felt overwhelmed by the abundance of guidelines and protocols related to IPC and experienced difficulties with selecting and applying the most relevant ones in their daily practice [12]. This review also supports the findings of the present study regarding facilitators as it has concluded that supportive work environments — including support from colleagues and support from the management team — play an important role in promoting IPC implementation [12]. The same review has suggested that clear communication and leadership, and the availability of resources were important factors sustaining IPC implementation in healthcare facilities. While this review was conducted in care settings other than disability care, it identified similar influences of micro, meso, and macro-level factors impacting IPC as our present study.

## Strengths and limitations

A strength of the present study is that the examination of perceived barriers and facilitators, as well as professional-reported recommendations, provides a solid and evidence-based foundation for the development of interventions that are better tailored to the opportunities, challenges and needs regarding IPC in disability care. Furthermore, this study demonstrates triangulation of both qualitative and quantitative data by incorporating both closed-ended and open-ended questions in our questionnaire. This approach enhances the robustness of our findings and strengthens the overall quality of the study [25]. Another strength of this study is its comprehensive examination of the barriers to and facilitators of IPC on multiple levels, encompassing micro, meso, and macro perspectives.

Although this study provides valuable insights into the perceived barriers to and facilitators of IPC in RCFs for people with IDDs, there are some limitations to be considered. Firstly, the questionnaire was created specifically for this study, thereby, it is important to note that it has not been validated. Nevertheless, our questionnaire was developed based on established theoretical frameworks, validated determinant measurements (such as the MIDI checklist) and insights from previous qualitative findings [11]. In addition, to enhance content validity, we conducted pilot testing and sought input from a multidisciplinary expert group. A second limitation is the use of convenience sampling to select participants, which could introduce selection bias as it is possible that more IPC-minded professionals were reached. Additionally, the recruitment method makes it challenging to accurately report the response rate since the number of professionals reached in the facilities is unknown. However, this study aimed to include a diverse group of professionals from various occupations, educational backgrounds, and layers of the organisation, which suggests that the sample was reasonably representative of the study population. Thirdly, as is inherent to questionnaire studies, it is crucial to recognise the possibility of social desirability bias influencing participants’ responses to the questionnaire [26]. Nonetheless, the findings of our study demonstrate a wide range of perspectives regarding perceived barriers and facilitators. Therefore, we anticipate that the influence of this potential bias on our results is relatively low. Moreover, it is important to note that while the organisation of disability care may vary among countries, we believe that fundamental issues with IPC in these care settings are likely to exhibit similarities across Western countries. Therefore, we anticipate that the insights into barriers and facilitators presented in our study hold relevance and applicability in an international context.

## Implications for practice

To improve IPC in disability care settings, it is crucial to develop effective strategies that address the barriers and enable the facilitators identified in this study. The findings of our study suggest that the client group can pose a challenge to IPC implementation. This underscores the importance of involving clients and their relatives in the planning of IPC improvement strategies and IPC implementation [27, 28], as also recommended by professionals. The involvement and engagement of patients in IPC improvement efforts have been identified as key strategies for promoting IPC adherence and implementation within facilities and reducing healthcare-associated infections [27, 28]. As this approach can help to increase awareness, understanding, and ownership of IPC practices among patients and their families. Specific interventions to overcome client-related challenges such as lack of IPC awareness or skills can include IPC education programmes that are especially aimed at clients, such as humorous hygiene lessons. In addition, stimulation and motivation of clients by professionals could enhance IPC compliance and cooperation [11]. Therefore, professionals are recommended to prioritise client participation to enhance their involvement and engagement. Nevertheless, as RCFs provide care to a diverse range of different client groups and accompanying care needs, these IPC improvement strategies need to be tailored to the unique needs and characteristics of the client population as the degree of intellectual impairment (and therefore potential understanding) may vary across different care settings [11, 18].

The identified areas of tension between IPC and the home-like environment suggest that efforts to improve IPC may need to address sectoral beliefs, values, and practices that conflict with IPC guidelines. This highlights the need for IPC policies and guidelines to be tailored to the local care context of disability care settings, taking into account the specific needs and challenges faced within each different care setting. An initial recommendation is that guidelines and protocols should include specific guidance on how to navigate dilemmas between IPC and the home-like environment. The development of a practical and context-specific IPC guideline is essential. Therefore, a guideline or protocol that is visualised as a decision tree may be a recommended approach [29]. This will provide a clear and concise visual representation of the decision-making process, allowing HCWs to easily understand the steps involved in IPC and navigate potential dilemmas between IPC and the home-like environment, which will ensure that HCWs are able to make informed decisions that align with IPC guidelines and the unique settings of disability care. Furthermore, a decision tree can visualise the most important steps in IPC, thereby providing a prioritisation of required actions and mitigating the overwhelming nature of existing protocols and guidelines. It is recommended to develop a decision-making tree for every specific local context of the disability care setting, as this enhances the relevance, applicability, and compatibility of the IPC guidelines with the workplace.

Given that social support among colleagues is identified as an important facilitator, encouraging social support among colleagues through interprofessional (peer) coaching can also be an effective IPC improvement strategy [30, 31]. This approach involves HCWs supporting and learning from each other through informal coaching sessions, sharing of experiences and knowledge, and feedback. Peer coaching — such as interprofessional coaching — can help to promote a culture of collaboration, communication, and continuous improvement, which may promote effective IPC [32]. For the implementation of interprofessional coaching programmes, it is important that efforts are ongoing and sustained to ensure the continuity of awareness, knowledge, and skills regarding IPC within a team, even in the face of staff turnover.

Reducing workload is another critical aspect of IPC improvement strategies in disability care settings. HCWs in these settings often experience high work pressure, as a result, facilities should aim to reduce workload by addressing factors that contribute to high work pressure, such as understaffing or high administrative workload. As reducing workload and work pressure is challenging in healthcare settings, strategies should be implemented for managing competing demands [33]. This can involve the discussion of challenges regarding IPC requirements in the context of high work pressure and developing creative solutions to overcome these challenges. By engaging in ongoing communication and collaboration, teams can work together to streamline IPC practices, identify areas where additional support may be needed, and ensure that IPC remains a priority even when facing competing demands. In addition, fostering an IPC culture including shared responsibility within the organisation is essential [12]. This involves establishing a culture of accountability and shared responsibility, and continuous improvement, where IPC practices are highly valued and seen as an integral part of quality care delivery. By creating an IPC culture, professionals can feel empowered to identify and address problems and share feedback and ideas for improvement [34]. Such a culture can help to ensure that IPC practices are consistently applied, monitored, and evaluated, which will contribute to the sustained implementation of IPC in RCFs for people with IDDs.

Our findings also suggest that bridging the gaps in education and training for new employees and non-medical professionals is important to enhance IPC practices. Moreover, on a care sector and policy level, promoting collaboration between disability care facilities and other health organisations (transdisciplinary collaboration), along with increased governmental action regarding IPC policies, legislation, and initiatives (e.g., including IPC as a structural component of the curriculum of basic educational programmes) may be important for comprehensive IPC implementation. These findings underscore the necessity of acting on multiple levels to achieve significant improvements in IPC within the disability care sector. The implementation of multilevel or multifaceted strategies has shown to be effective in promoting and sustaining IPC [35, 36].

## Conclusions

To improve IPC in disability care settings, it is crucial to develop effective strategies that address the multilevel challenges and opportunities identified in this study. Based on our main findings, IPC improvement strategies should prioritise the involvement of clients and their relatives, as well as focus on developing practical and context-specific IPC guidelines. Additionally, promoting social support among colleagues through interprofessional coaching, reducing workload, and fostering an IPC culture including shared responsibility within the organisation are important approaches towards optimally implementing and sustaining IPC in these settings.

### Electronic supplementary material

Below is the link to the electronic supplementary material.

Supplementary Material 1

## Data Availability

The datasets used and analysed during the current study are available from the head of the data-archiving of the Public Health Service South Limburg on reasonable request. Interested researchers should contact the head of the data-archiving of the Public Health Service South Limburg (Helen Sijstermans: helen.sijstermans@ggdzl.nl) when they would like to re-use data.
