# Supplementary material for: Perceived barriers and facilitators to infection prevention and control in Dutch residential care facilities for people with intellectual and developmental disabilities: a cross-sectional study
Source: BMC Public Health. 2024 Mar 5;24:704. doi: 10.1186/s12889-024-18159-9 (PMC10916042; doi:10.1186/s12889-024-18159-9)
Supplement: Supplementary file 1 — Supplementary Material 1 [file 12889_2024_18159_MOESM1_ESM.docx]

**Additional file 1: Perceived barriers and facilitators to IPC implementation, reported by professionals from residential care facilities (RCFs) for people with intellectual and developmental disabilities (IDDs), categorised by corresponding level**

| **Perceived barriers** | **proportion (n) of participants who reported the barrier or facilitator (agree-totally agree)** |
| --- | --- |
| *Guideline level* |  |
| Complexity – lack of comprehensibility of IPC guidelines | 9.7% (29) |
| Complexity – information overload in IPC guidelines | 25.9% (77) |
| Feasibility – overwhelming quantity of IPC guidelines | 32.9% (97) |
| *Client level (as reported by the professional)* |  |
| Lack of hygiene awareness among clients | 63.5% (195) |
| Lack of cooperation from clients with IPC | 28.6% (84) |
| Lack of IPC skills among clients | 62.8% (189) |
| Difficulties with IPC implementation among clients who are predominantly healthy or have fewer care needs | 45.2% (123) |
| Difficulties with IPC implementation due to diversity in client groups | 61.9% (182) |
| *Organisational level* |  |
| Staff shortages | 20.6% (63) |
| High staff turnover | 28.5% (88) |
| High work pressure | 38.9% (121) |
| Lack of IPC material resources and facilities | 14.4% (45) |
| Lack of financial resources for IPC | 5.8% (17) |
| *Care sector level* |  |
| Competing values between IPC and the home-like environment | 42.1% (123) |
| **Perceived facilitators** |  |
| *Guideline level* |  |
| Availability of IPC guidelines | 81.8% (243) |
| Accessibility of IPC guidelines | 69.8% (215) |
| Procedural clarity – clear and specific descriptions in IPC guidelines | 83.1% (246) |
| Compatibility – fit of IPC guidelines with working method | 50% (147) |
| Adaptability – flexibility to adjust or customise IPC guidelines | 37.8% (109) |
| *Interpersonal level* |  |
| *Professional interaction (team)* |  |
| Mutual professional feedback and accountability regarding IPC | 60.2% (192) |
| Feedback from supervisor on IPC | 40.8% (130) |
| Exemplary behaviour of colleagues regarding IPC | 63.6% (203) |
| Perceived social support from colleagues on IPC | 89.7% (286) |
| Perceived social support from supervisor on IPC | 80.3% (256) |
| Interprofessional collaboration regarding IPC | 49.7% (145) |
| *Professional-client interaction* |  |
| Exemplary behaviour of professionals towards clients regarding IPC | 66.8% (193) |
| Feedback and accountability of professionals towards clients regarding IPC | 49.8% (141) |
| Stimulation and motivation of professionals towards clients regarding IPC | 43.1% (122) |
| *Organisational level* |  |
| Educational system  IPC education and training for non-medical professionals  IPC education and training for new employees  IPC education and training for clients | 42.5% (111)  44.4% (126)  27.6% (76) |
| Managerial support/support board of directors for IPC | 63.2% (177) |
| Priority for IPC | 66.4% (198) |
| Structural attention to IPC | 61.4% (186) |
| Organisational sense of urgency for IPC | 73.7% (216) |
| Internal change agents for IPC | 60.1% (170) |
| Professional responsible for IPC (e.g., infection control professional) | 55.4% (158) |
| *Care sector level* |  |
| IPC as professional norm in the disability care sector | 60.5% (173) |
| IPC as collective concern in the disability care sector | 48.4% (125) |
| Sufficient collaboration between disability care facilities (intersectoral collaboration) regarding IPC | 43.4% (102) |
| Sufficient collaboration between disability care facilities and other health organisations such as the Public Health Service or hospitals (transdisciplinary collaboration) regarding IPC | 50.6% (120) |
| *Policy level* |  |
| Sufficient information provision from governmental agencies on IPC | 62.6% (179) |
| Sufficient information provision from professional association on IPC | 51.2% (124) |
| Sufficient governmental initiatives and policies regarding IPC | 47.1% (114) |
| Abbreviations. IPC=infection prevention and control. | |
